# Supplementary material for: Cervical cancer screening in a population of black South African women with high HIV prevalence: A cross-sectional study
Source: PLOS Glob Public Health. 2022 Nov 15;2(11):e0001249. doi: 10.1371/journal.pgph.0001249 (PMC10021232; doi:10.1371/journal.pgph.0001249)
Supplement: Attachment — Submitted filename: Response to reviewers.docx [file pgph.0001249.s001.docx]

| **Response to reviewers** | | |
| --- | --- | --- |
| **Comments** | **Amendments** | **Page/line number** |
| Please amend your detailed online Financial Disclosure statement. This is published with the article. It must therefore be completed in full sentences and contain the exact wording you wish to be published.  State what role the funders took in the study. If the funders had no role in your study, please state: “The funders had no role in study design, data collection, and analysis, decision to publish, or preparation of the manuscript.” | The financial disclosure has been amended to”  “This study was co-funded by the National Research Foundation grant 93030 for Olalekan Ayo-Yusuf and the Project SHPC 000 fund of the Department of Family medicine, the University of the Witwatersrand for Olufemi Omole. The funders had no role in the study design, data collection, and analysis, decision to publish, or preparation of the manuscript”. | Page 13, Line 332-335 |
| Please update your online Competing Interests statement. If you have no competing interests to declare, please state: “The authors have declared that no competing interests exist.” | The competing interest statement has been updated to:  “The authors have declared no competing interests in this study” | Page 16, Line  330 |
| In the online submission form, you indicate that your data is not available for proprietary reasons and have provided a contact point for accessing this data. Please note that your current contact point is a co-author on this manuscript. According to our Data Policy, the contact point must not be an author on the manuscript and must be a third party. Please revise your data statement to a non-author institutional point of contact, such as a data access or ethics committee, and send this to us via return email. Please also include contact information for the third-party organization, and please include the full citation of where the data can be found. | Our ethics agreement with participants was that the data would only be accessible to the study team due to the sensitive information contained in the data; hence, it would compromise the HREC ethical standards to allow the data to be publicly available in a public repository, within the manuscript itself or uploaded as supplementary information. We are happy to share the data, or parts of the data, on a case-by-case basis. Please contact Joel Francis (non-author), for such requests at Joel.francis@wits.ac.za |  |
| Please provide separate figure files in .tif or .eps format and ensure that all files are under our size limit of 10MB.  For more information about how to convert your figure files please see our guidelines: <https://journals.plos.org/globalpublichealth/s/figures> | Figures have been formatted accordingly |  |
| Please change the item type of Figure 1 from 'Supporting Information' to 'Figure'. | The item type of Figure 1 has been changed from 'Supporting Information' to 'Figure 1 ' |  |
| Please include a legend for Figure 1 in your manuscript | A legend for figure 1 has been included |  |
| We have noticed that you have uploaded Supporting Information files, but you have not included a list of legends. Please add a full list of legends for your Supporting Information files after the references list. | A full list of legends for Supporting Information files is included after the references list. | Page 22-23  Lines 464-480 |
| The study aim has clearly been articulated, apart from the inclusion criteria, “based on the high yield Gauteng report of 2013/2014”, the authors did not clearly specify the inclusion/exclusion criteria (e.g risk, stage of disease progression, women on chemotherapy already etc.) with sufficient detail and all necessary information critical to the study | We recruited consecutive women who presented to the cervical screening programs of PHC clinics across the five health districts in Gauteng province – Johannesburg, Tshwane, Ekurhuleni, Sedibeng, and West Rand, between 2017 and 2020. During the study period were eligible and approached by a trained research assistant to participate at the vital signs station. The research assistants explained the nature and objective of the study to each woman and in addition, gave a participant information leaflet. Further explanations and clarifications were offered as needed. Those who indicated an interest in the study were directed to a private room where written informed consent was obtained, and the questionnaire administered. Thereafter, participants were taken to the nurse who then performed the cervical smear as per usual care or if not yet time, back to their positions on the service queue. Recruitment of participants continued until 40 participants were recruited in each clinic. Patients who were too ill and pregnant were excluded from the study. | Page 8-9, lines 147-161 |
| Line 128-129 the authors state that “The facilities were purposefully selected for their high yields in cervical cancer screening in the 2013/14 Gauteng clinics report”, what was the reason for not including low-moderate yield sites? Could they have been selectively excluded yet they could have been that way because of the insufficient information from primary care providers, negative community opinions relating to the procedure, and fear of having an HIV test at the same time? This needs to be articulated with clarity. | We acknowledge the error. The eligible facilities were randomly selected from the list of all facilities in the 5 districts. We looked at the 2013/14 Gauteng clinics report, which gave us percentages of contribution to the total sample. Participants from the selected facilities were consecutively recruited from all the women visiting the facilities for routine Pap smears. | Page 7-8, lines 125-145 |
| In lines 40-141, the authors state that 749 women were recruited, but only data for 672 were extracted for this study. What happened to the 77 who were excluded from the analyses? Please account for them. | A total of 749 women were recruited for the primary objective. 77/749 (11%) were excluded from this analysis because this study focused on those women who self-identified as blacks. Black women were included because they are patrons of public health services in South Africa. Furthermore, other races were excluded from the study because their numbers were too small to provide a reliable estimate related to the objectives of the study. | Page 9 lines 175-177 |
| Overall, there is no mention of the sample size. How did you justify your sample size? Please explain how the study size was arrived at | The clinics were randomly selected from each district cluster. The number of clinics per district was proportional in respect to the proportion each district contributed to the total provincial Pap smear count for 2013/14. Considering the primary objective of the main study, we estimated a minimum sampdle of 1032; assuming a 95% confidence level, 80% power, 8.4% SLT use prevalence among women and a 6.5% incidence rate of abnormal Pap smears. We adjusted upwards by 10% for inadequate smears and/ or incomplete or lost data, resulting in a sample size of 1135. Furthermore, to reduce the effects of intra and inter cluster correlations (ICC), we calculated the Effective sample size (ESS) using the equation: ESS= mk / [1+p(m-1)], where: 1+p(m-1) equals the design effect (DE), K equals the number of clinic cluster, ESS equals the effective sample size, m equals the number of participants per cluster / clinic and p equals the ICC coefficient. Assuming an ICC coefficient (p) of 0.01 and an ESS of 1135, an m of 40 participants per clinic and a total of 40 clinics (k) across the 5 districts of Gauteng province were determined and a final sample size of 1600 – forty (40) to be recruited across Johannesburg (17 clinics), Tshwane (7 clinics), Ekurhuleni (11 clinics), Sedibeng (2 clinics), and West Rand (3 clinics). However, data collection was halted due to the COVID-19 pandemic restrictions and only 749 participants were recruited across 20 clinics in the five districts – Johannesburg (315), Ekurhuleni (170), Tshwane (79), West Rand (105) and Sedibeng (80). | Page 7-8, lines 125-145 |
| The ‘recruitment and participants’ section are not clear. | We recruited consecutive women who presented to the cervical screening programs of PHC clinics across the five health districts in Gauteng province – Johannesburg, Tshwane, Ekurhuleni, Sedibeng, and West Rand, between 2017 and 2020. During the study period were eligible and approached by a trained research assistant to participate at the vital signs station. The research assistants explained the nature and objective of the study to each woman and in addition, gave a participant information leaflet. Further explanation and clarifications were offered as needed. Those who indicated an interest in the study were directed to a private room where written informed consent was obtained, and the questionnaire administered. Thereafter, participants were taken to the nurse who then performed the cervical smear as per usual care or if not yet time, back to their positions on the service queue. Recruitment of participants continued until 40 participants were recruited in each clinic. Patients who were too ill and pregnant were excluded from the study | Page 8, lines 147-161 |
| Was the selection process used likely to select participants that were representative of the population under investigation? Please clarify | A total of 749 women were recruited for the primary objective. 77/749 (11%) were excluded from this analysis because this study focused on those women who self-identified as blacks. Black women were included because they are patrons of public health services in South Africa. Furthermore, other races were excluded from the study because their numbers were too small to provide a reliable estimate related to the objectives of the study. | Page 9 lines 175-177 |
| The authors should describe any efforts to address potential sources of bias | The study was based on self-reports and therefore has potential for social desirability. This was addressed by the openness, transparency, and non-threatening engagement of the trained field workers with participants.  The study also had the potential of information bias since study participants were women who had come to the clinics for cervical cancer screening of their own volition, they were more likely to report better knowledge and screening behaviors than the larger clinic women cohort or those who had not visited the clinic. The true prevalence of screening in the past 10 years among black women in the general population would therefore be probably less than that reported in this study | Page 15-16, lines 307-318 |
| What measures did the authors undertake to address and categorize non-responders? Please describe this | No information was collected from those who did not consent to participate; therefore, we could not categorize them.  Not everybody responded to every variable, this is indicated in tables 1 to 6 |  |
| Line 144-145 the authors state that “The measurement tool was a semi-structured, research-administered questionnaire developed de novo based on the literature (31-35)” how did the authors correctly measure the risk factors and outcome variables using a measurement tool that had not been trialed, piloted, or published previously? This is not clear please clarify | The tool was piloted in 1 clinic that did not form part of the study | Page 9, lines 164-166 |
| Line 160-164 the authors mention using bivariate logistic regression models. However, there is no description of all the statistical methods used including those used to control for confounding. Can you include this too? | Associations between the cervical cancer screening uptake and sociodemographic characteristics, relevant sexual behaviour, HIV status, tobacco use, history of STI, knowledge of the risks of cervical cancer, and sources of information on cervical cancer and screening were explored using bivariate analysis. All significant exposure variables were included into the multivariable logistic regression model. We reported adjusted odds ratios, their corresponding 95% confidence intervals and p values. A p-value <0.05 was considered statistically significant | Page 10, lines 184-190 |
| Describe any methods the authors used to examine subgroups and interactions | There was no study objective that necessitated examining interactions (moderation effects). However, subgroups were examined in bivariate analysis (table 3) |  |
| The authors need to explain how missing data were addressed | Missing data have been added to the tables (see tables 1 to 6) | Tables 1-5 |
| The authors should describe any sensitivity analyses done | Sensitivity analyses were not applicable in this study |  |
| **Results** |  |  |
| In Table 1, apart from the marital status variable, all the other variables do not each total to 672 as stated by the authors, and yet the percentages total 100. Please revisit your numbers and report the correct ones | This has been addressed on the tables, missing data has been added |  |
| The authors should report numbers of individuals at each stage of the study e.g., numbers potentially eligible, examined for eligibility, confirmed eligible, included in the study | Based on the estimated sample size, 1600 participants were potentially eligible for the study. The records for those who did not meet the eligibility criteria to be included in the study were not kept, therefore the number of those examined for eligibility could not be established, and 749 participants were confirmed as eligible for the study. Out of 749 women who participated, 672 met our inclusion criteria for our study. Only 77/749 (11%) were excluded because they followed the exclusion criteria. |  |
| They should also give reasons for non-participation at each stage | Only those who met the inclusion criteria and consented to participate were included  the records were not kept for those who did not meet the eligibility criteria to be included in the study  Only 77 participants were excluded from the analysis of this study as they were non-blacks. |  |
| Line 179-180 the authors state “only 42.4% of participants reported screening for cervical cancer in the last 10 years” drawn from Table 2. However, this is not clear in Table 2. Please clarify | The prevalence of cervical cancer screening in the last 10 years uptake is reported in the table 1 |  |
| Line 183-184 is incomplete. Where did the rest obtain information on cervical cancer screening from | Currently, we only have information for 637/672 participants, and 35 are missing therefore we do not have data regarding their source of information. |  |
| **Other minor comments**: • There are a few typos to review. e.g., line 56-58, line 83, | Typos have been corrected |  |
| Line 149, HIV status and use of antiretroviral drugs is not part of gynecological information. | The statement is corrected |  |
| Data Availability Statement. | Our ethics agreement with participants was that the data will only be available to the study team; hence it would compromise the HREC ethical standards to allow the data to be publicly available in a public repository, within the manuscript itself or uploaded as supplementary information. We are happy to share the data, or parts of the data, on a case-by-case basis. Please contact Joel Francis (non-author), for such requests at Joel.francis@wits.ac.za |  |
|  |  |  |
| **Reviewer 3** |  |  |
| Is the 40 women per health facility something computed? Please add further details on the original sample size calculation | The clinics were randomly selected from each district cluster. The number of clinics per district was proportional in respect to the proportion each district contributed to the total provincial Pap smear count for 2013/14. Considering the primary objective of the main study, we estimated a minimum sample of 1032; assuming a 95% confidence level, 80% power, 8.4% SLT use prevalence among women and a 6.5% incidence rate of abnormal Pap smears. We adjusted upwards by 10% for inadequate smears and/ or incomplete or lost data, resulting in a sample size of 1135. Furthermore, to reduce the effects of intra and inter-cluster correlations (ICC), we calculated the Effective sample size (ESS) using the equation: ESS= mk / [1+p(m-1)], where: 1+p(m-1) equals the design effect (DE), K equals the number of clinic cluster, ESS equals the effective sample size, m equals the number of participants per cluster/clinic and p equals the ICC coefficient. Assuming an ICC coefficient (p) of 0.01 and an ESS of 1135, an m of 40 participants per clinic and a total of 40 clinics (k) across the 5 districts of Gauteng province was determined and a final sample size of 1600 – forty (40) to be recruited across Johannesburg (17 clinics), Tshwane (7 clinics), Ekurhuleni (11 clinics), Sedibeng (2 clinics), and West Rand (3 clinics). However, data collection was halted due to the COVID-19 pandemic restrictions and only 749 participants were recruited across 20 clinics in the five districts – Johannesburg (315), Ekurhuleni (170), Tshwane (79), West Rand (105), and Sedibeng (80). | Page 7-8, lines 125-145 |
| Please share the data collection tool in the appendix | The data collection tool is attached |  |
| **For data analysis**: - Please do not write STATA. It is Stata and please cite. | Citation for STATA has been added. |  |
| Line 162. It is multivariable logistic regression. Or multiple logistic regression Line 161 it is stated that only statistically significant on bivariate pass to the multivariable. Looking at your results on the table 3 it doesn't seem to be this  I see a lot of characteristics on the table 3 that are not on table 1 or 2. Why? | Associations between the cervical cancer screening uptake and sociodemographic characteristics, relevant sexual behaviour, HIV status, tobacco use, history of STI, knowledge of the risks of cervical cancer, and sources of information on cervical cancer and screening were explored using bivariate analysis. All significant exposure variables were included into the multivariable logistic regression model. We reported adjusted odds ratios, their corresponding 95% confidence intervals, and p values. A p-value <0.05 was considered statistically significant | Page 10, lines 184-190 |
| For the screening prevalence, it would be good to report the 95% confidence interval (in the abstract as well as in the main manuscript). So I would recommend changing table 2: - Remove the total for each variable. This is unnecessary. - Right now the sequence of the columns is "characteristics, N, % (for No), N, % (for Yes), N (for total) and p-value. I propose to remove the N for total.). | All tables have been amended according to the suggestions. Totals for each variable in all tables have been removed and the 95% confidence interval is reported in table 3 |  |
| Make the percentages per column; remove the p-values; Add a column for prevalence of screening (and its confidence interval | The prevalence of cervical cancer screening in the last 10 years uptake is reported in the table 1 |  |
| **Table 3:** - Remove the N and %. That is on table 2. | The N and % are removed from table 2 |  |
| - For marital status please use te single or married as reference. | The variable “single” is used as a reference instead of divorced (Table 3) |  |
| - What 1< means on the number of lifetime sex partners | The variable has been removed |  |
| **Table 4:** - Please remove the subtotals per variable. Just add an overall row stating that the total is 666 | Sub totals have been removed from table 4 and add missing data has been included |  |
| **Table 5:** - The same comments as for table 4. The overall total here is 488. | The Sub totals have been removed from table 5 and add missing data has been included |  |
|  |  |  |
| **Reviewer 4** |  |  |
| Attending screening is complex, because it is a process rather than a single event. For example, after the screening procedure women have to receive the test results and if positive return for treatment, before they return for follow-up and re-screening. In reality, several of these steps may fail and although woman may attend the screening procedure, the overall outcome may actually fail, if the woman e.g. did not undergo treatment of the precancerous lesion. In the I-SAM model this level of complexity is not well addressed (to my understanding) and the authors may want to include this in their discussion. In this regard, the WHO Elimination Strategy targets to not only screen 70% of women but also to treat 90% of those with pre-cancer. The authors should consider to make reference to the WHO CC Elimination Strategy. | Limitation  The study was not able to account for how many women who were screened came for treatment according to the I-SAM model  I-SAM model has been incorporated into the discussion | Page 15-16, lines 307-318  Page 15-24, lines 294-305 |
| Please follow all steps of the STROBE guideline for reporting, including a report on the number of participants potentially eligible, screened for eligibility, finally included, as well as missing data for the different variables in the table in there are any. From the STROBE checklist o a) Report numbers of individuals at each stage of study—eg numbers potentially eligible, examined for eligibility, confirmed eligible, included in the study, completing follow-up, and analysed o (b) Give reasons for non-participation at each stage o (c) Consider use of a flow diagram  o (b) Indicate number of participants with missing data for each variable of interest | STROBE guideline is attached |  |
| Please add total number of participants to table 1. | The total number of participants in table 1 has been added. |  |
| Please revise references in the text, the references should be set in front of the punctuation, not behind | References corrected |  |
