# Supplementary material for: Cervical cancer screening in a population of black South African women with high HIV prevalence: A cross-sectional study
Source: PLOS Glob Public Health. 2022 Nov 15;2(11):e0001249. doi: 10.1371/journal.pgph.0001249 (PMC10021232; doi:10.1371/journal.pgph.0001249)
Supplement: Attachment — Submitted filename: Response to reviewers.docx [file pgph.0001249.s002.docx]

**Response to reviewers**

| **Comment** | **Amendment** | **Page/line number** |
| --- | --- | --- |
| **Reviewer 1** | | |
| I**ntroduction** | | |
| Line 149, include the month of the year in your statement “between 2017 and 2020” | Months are included in the statement(March 2017- April 2020) | Page 8, line 149 |
|  |  |  |
| **Results** | | |
| In Table 1, your percentages on “cervical cancer screening in the last 10 years does not add up. Those who said yes, no and missing total 672, while the individual percentages do not total 100 % (42.3 + 57+ 0.07) what happened to 0.63 %? | In Table 1, percentages on “cervical cancer screening in the last 10 years has been adjusted | Pages 23-25 |
|  |  |  |
| Table 1 percentages are wrong and misleading, some exceed a sum of 100% or are far less than 100% for example education status, marital status, ever treated for STD, current sexual partner, number of lifetime sexual partners etc. please check your figures again and update them accordingly. | All the percentages in Table 1 have been adjusted accordingly | Pages 23-25 |
|  |  |  |
| Line 166 -172; Using the de novo questionnaire tool, to capture information on “The questionnaire collected information on 167 participants’ demographics (age, level of education, occupational status, and marital status), 168 tobacco use (patterns of cigarette smoking, exposure to second-hand smoke…….” Can the authors explain why in table 2 they state N=672, and go on to put a disclaimer in line 467 that the totals varied because of missing values? Was this tool validated correctly? | All variables had missing values except marital status. The total number of participants was 672.  The disclaimer in line 480 has been adjusted accordingly | Page 27  Line 480 |
|  |  |  |
| **Reviewer 2** |  |  |
| Please see the official documentation of Stata. It is a very common mistake to write STATA, as if it was an acronym. Please correct to be Stata. | STATA is changed to Stata | Page 9  Line 175 |
|  |  |  |
| Ok, the adjusted regression results. But you do not have to keep only bivariate significant factors. As long as you justify in the methods that, for example, you kept HIV regardless of the p-value. | Comment noted |  |
|  |  |  |
| **Second paragraph of the introduction** |  |  |
| Citation 6 - please find an additional reference to this. And please provide an URL (link) for citation 6. | The reference is replaced by Meites, E., Gee, J., Unger, E., & Markowitz, L. (2007). Human Papillomaviruses. |  |
|  |  |  |
| Lines 54 and 55, in the among the best possible protection against cervical cancer. Why the vaccine is not included here? | Vaccination has been added | Page 4  Line 54 |
